# Customer Restaurant Choice: An Empirical Analysis of Restaurant Types and Eating-Out Occasions

**DOI:** 10.3390/ijerph17176276

**Published:** 2020-08-28

**Authors:** Bee-Lia Chua, Shahrim Karim, Sanghyeop Lee, Heesup Han

**Affiliations:** 1 Department of Food Service and Management, Faculty of Food Science and Technology, Universiti Putra Malaysia, Serdang 43400, Malaysia; chuabeelia@upm.edu.my (B.-L.C.); shahrim@upm.edu.my (S.K.); 2 Major in Tourism Management, College of Business Administration, Keimyung University, Daegu 42601, Korea; leesanghyeop@kmu.ac.kr; 3 College of Hospitality and Tourism Management, Sejong University, Seoul 143-747, Korea

**Keywords:** restaurant choice, restaurant types, eating-out occasions, descriptive analysis, key restaurant selection factors, ranking

## Abstract

This study investigated restaurant customers’ perceived importance of key factors in accordance with dining occasions and restaurant segments. Our investigation into restaurant selection and situational factors present two types of empirical evidence regarding customers’ choice of restaurant. First, menu price was customers’ top priority in restaurant selections for full-service, quick-casual, and quick-service restaurants. Second, restaurant customers rated the importance level of restaurant selection criteria differently according to eating-out occasions. The importance of menu price was greatest for both quick meal/convenience and social occasion, brand reputation was the most important factor for business necessity, and word-of-mouth recommendation was greatest for celebration.

## 1. Introduction

In today’s competitive restaurant business, an increase in restaurant business competition implies that customers nowadays have more dining choices to choose from than ever before, ranging from fast food to fine dining restaurants [1,2]. As a result, customer expectations of restaurant offerings are ever-increasing, and they are now more demanding in choosing better restaurant choices based on what they can get from their decision [3]. In view of the growing phenomenon toward eating-out, knowledge of the criteria used by customers in the selection of a restaurant is strategic in understanding food consumption trends [4]. In fact, as digital technology continues to advance, it is becoming increasingly challenging to please restaurant customers as their eating-out behavior is now more sophistically evolved, and they are cognizant of the customer value [5,6,7]. Thus, it is particularly important that restauranteurs stay on top of consumer behavior in the restaurant industry so that they can cater to the needs and wants of customers appropriately. This present study overcame this challenge by addressing the following research questions: (1) What is the relative importance of a restaurant selection factor in relation to other factors? (2) How do key factors in restaurant selection differ across eating-out occasions? (3) How do key factors in restaurant selection differ across restaurant segments? 

A restaurant customer’s decision-making process begins when he/she recognizes a need that can be fulfilled by consuming the products/services offered by a restaurant [8]. The need for restaurant consumption may be driven by various factors, such as having quick meals, celebrating special occasions, entertaining business clients, etc. Customers will search for relevant information about restaurants, compare restaurant options, and make the final purchase decision of which restaurant to dine at [9]. The theory of information integration [10] posits an individual’s overall attitude toward a product/service is mutually shaped by the perceived actual performance and the perceived importance of the product/service. In hospitality business, it is essential that service firms understand how important each product/service’s key factor is in customers’ decision making. While service firms can operationally control a product/service’s performance, customers, the direct receivers of a product or service, primarily determine the importance of a product/service’s decisive factors [11,12,13]. Hence, several marketing scholars have investigated the importance of key factors in customer decision making across hospitality and tourism backgrounds, such as hotel [14], cruise [15], and destination [16].

A review of past research on restaurant management reveals that the factors driving customers’ choice of restaurant are price, food, variety, reputation, promotion, location, and information sources [8,17,18,19,20,21]. In this regard, the key factors in restaurant selection have relevance only if they are being perceived as significantly important from the viewpoints of customers. Restaurateurs often make costly expenditures on marketing activities to attract customers by utilizing various marketing techniques from menu development to sales promotion. However, any change in marketing activities meant to expand the customer base and increase sales requires concrete and sound evidence to evaluate whether such efforts payoff. Despite substantial interest in consumer behavior and restaurant marketing research among hospitality scholars [22,23,24,25], evidence of customers’ perceived importance of restaurant selection factors and how they vary across situational factors (i.e., dining occasions and restaurant segments) are surprisingly scant. Restauranteurs are left with little evidence on how restaurant choice factors influence customers’ eating-out decision making. When making an eating-out decision, customers often view a restaurant in terms of a set of characteristics that make it desirable, assigning an importance score to each factor [26]. Restauranteurs thus need to be mindful of whether a decisive factor is perceived by customers as generally important, or important depending on the context and situation, or if the factor is perceived to be trivial no matter what the context and situation. The effectiveness of restaurant marketing strategy can possibly be strengthened by discerning customer perception of important factors when making an eating-out decision. Of special relevance to this study, we theorized that the factors driving customers’ choice of restaurant vary with the occasion of eating-out as well as with the type of restaurant. Restaurant reputation, for example, may appeal to those who are planning for special occasions, such as a birthday or a wedding anniversary, rather than for those who want to eat-out simply to satisfy hunger. On the other hand, location may be perceived to be more important for quick-service restaurants than full-service restaurants. More accurate evidence, however, is needed.

Understanding how key factors driving customers’ choice of restaurant differ is critical to the continued advancement of customer decision-making knowledge and effective restaurant marketing strategies. First, while numerous studies in hospitality literature have explored the factors and attributes affecting restaurant customers’ decision to choose a restaurant, they have particularly focused on a restaurant segment, omitting the moderating variables when examining the attributes. Furthermore, previous studies have reported that there is a gap in the hospitality literature with respect to the understanding of drivers in customers’ eating-out decision making, and this situation has called for further investigation into the topic [3,27]. The present study attempted to bridge the literature gap by incorporating dining occasions and restaurant segments to better explain the underlying reason behind customers’ decision-making in the restaurant industry, and hence complement past research findings. We provided a picture regarding restaurant customers’ perceived importance of key factors in accordance with dining occasions and restaurant segments, which is the theoretical contribution of this study. Therefore, we expect that this present study would extend the customer decision-making literature. Second, from a practical viewpoint, an investigation of key factors driving customers’ restaurant choice in eating-out decision making not only can help restaurateurs understand restaurant customer perception of key factors when selecting a restaurant, but also form appropriate marketing strategies to attract existing and potential customers and outperform competitors. 

This study aimed to conduct an empirical research associated with critical factors for customers’ restaurant choice in the current restaurant industry using a descriptive analysis. The specific research objectives are as follows:

- The first objective was to rank the factors that are important for the selection of restaurants (i.e., (a) word-of-mouth recommendations from people I know, (b) online reviews from customers, (c) brand reputation, (d) brand popularity, (e) personal or past experience with the restaurant, (f) variety of menu items, (g) menu price, (h) sales promotion, and (i) location).
- The second objective was to uncover the order of importance among the factors for customers to consider when choosing a restaurant by eating-out occasions ((a) quick meal/convenience, (b) social occasion, (c) business necessity, and (d) celebration).
- The third objective was to identify the relative importance among the restaurant selection factors by restaurant types ((a) full-service restaurants, (b) quick-casual/convenience restaurants, and (c) quick-service restaurants).

## 2. Literature Review

### 2.1. Critical Restaurant Selection Factors

Attribute importance is the significance of an attribute for a product/service [28,29]. Customers typically evaluate product/service attributes that are perceived to be important in the purchase decision by assigning weight to each attribute in the product/service evaluation [30]. This relative importance of the attributes is decisive criteria often used by customers in comparing the product/service options, thus leading to purchase behavior [11,31]. In a similar vein, the importance of restaurant selection factors plays a crucial role in affecting customers’ restaurant choice. Based on the existing empirical studies, this study derived nine restaurant selection factors that are likely to affect customers’ decision in choosing a restaurant: word-of-mouth, online customer review, brand reputation, brand popularity, personal (past) experience, menu variety, menu price, sales promotion, and location. It is important to note that we did not include the core elements of restaurant operations: food quality (e.g., taste), service quality, and restaurant physical environment as they have been consistently and intuitively demonstrated to be highly important for restaurant survival [32]. The nine restaurant selection factors in our study, on the other hand, represent the value-added elements that can positively contribute restaurant business growth. The following sub-sections describe the determinants of customers’ restaurant choice.

#### 2.1.1. Word-of-Mouth Recommendations from People I Know

In the marketing literature, word-of-mouth refers to person-to-person communication about a product, a service, or a brand between a non-commercial communicator and a message receiver [28,33]. Word-of-mouth communication has been well-recognized as an influential drive in attracting new consumers and shaping customer behavior [33,34]. It is a communication process that allows people to share information about an offering which could either encourage or discourage potential customers to make a purchase. In fact, personal sources of information, including recommendations from family and friends, are perceived to be more reliable than commercial advertising media, and thus are more likely to induce customer’s positive/negative attitude towards a brand [35,36]. Sundaram, Mitra, and Webster [37] identified in their study that people involved in positive word-of-mouth for altruistic, product involvement, and self-enhancement reasons and in negative word-of-mouth for altruistic, anxiety reduction, vengeance, and advice-seeking reasons. In the service industries, such as restaurants and hotels, because consumers lack objective means of evaluating services, they typically depend upon subjective evaluations from family, friends, or acquaintances [35]. Because consumers may not know a restaurant (e.g., the food quality, service, environment, price) before actual consumption, they may seek referrals from an experienced source. For example, when seeking a nice restaurant for a celebration occasion, consumers will often ask friends for recommendations. Consistent with Stokes and Lomax [38], this present study viewed word-of-mouth as an informal and interpersonal communication of a restaurant between a customer and his/her acquaintance(s), of which such communication is independent of commercial influence.

#### 2.1.2. Online Review from Customers

The ever-increasing growth of Internet applications in hospitality has contributed to a great number of consumer-generated online reviews on different interactive forums. The importance of online reviews has been widely recognized in the hospitality marketing literature [39]. The customer decision-making process is strongly affected by online customer reviews posted on online review websites [40]. Put simply, online customer review websites are Internet channels that connect customers with many other customers. Online consumer reviews serve two functions [41]. First, it delivers information about a product/service. Second, it serves as a recommendation. As communication technology evolves, the role and significance of online reviews have been further heightened as people can make their opinion about and give feedback on a product/service easily available to other consumers [42]. Online review is particularly relevant for service-oriented products, such as hospitality products. Given the absence of tangibles, people often look to the tangible clues of the service to assist them in making a decision [35]. Online reviews primarily derive from many users who discuss and give insight into specific products/services to others [43]. Online reviews made by other customers about product and service performance appear to provide a clue as to whether the target brand can be trusted [44]. It also has been found to reduce consumers’ perceived risk and uncertainty prior to actual consumption [45]. Undeniably, consumers are increasingly relying on online search and review engines when making purchase decisions [46]. These online reviews are likely to encourage or detract potential customers from using a brand [40]. While some studies demonstrated that online reviews could reduce cognitive loads of consumers and thus are likely to result in increased sales [47], some studies reported that online reviews are perceived as having lower trustworthiness than traditional word-of-mouth due to the absence of source cues on the Internet [48]. Examining the relative importance of online reviews in restaurant customers’ decision-making would be useful for restauranteurs to better understand the significance of online reviews on their business. 

#### 2.1.3. Brand Reputation of Restaurant

Brand reputation reflects a mixture of reliability, admiration, benevolence, respect, and confidence of a brand [49]. It is a signal for the underlying quality of a company’s product or service offerings to customers [50]. A well-known reputation is psychologically easier for customers to choose a brand over another [51]. A reputable brand conveys a psychological assurance of the brand quality, thus creating customer trust [52]. Consumers typically have more trust in a brand if the brand has a favorable reputation as a result of consistently excellent performance [53]. Another stream of logic that lends support to the role of brand reputation is its influence on customers’ confidence in assessing a brand quality [54]. Stated differently, customers’ level of uncertainty can be reduced by choosing a reputable brand. In addition, brand reputation serves as a precursor of customer loyalty [55,56]. The influence of brand reputation on customer loyalty is in accordance with signal theory where consumers tend to associate themselves with brands of high reputation as part of self-enhancement [57]. In tandem with the positive correlation between brand reputation and brand quality, a restaurant’s reputation could be a critical consideration for customers when choosing a restaurant [58]. Recognizing the fact that consumers are likely to rely on reputation to infer restaurant quality, restaurant operators tend to devote efforts and utilize resources to develop a brand reputation [59]. Drawing upon this discussion, we suggest that brand reputation can add value to a restaurant’s brand equity [60], which is likely to influence customers’ decision-making.

#### 2.1.4. Brand Popularity

In general, brand popularity measures the extent to which a brand is broadly consumed by customers. This decisional tool has an information processing advantage by which a consumer can lessen his/her cognitive efforts in making purchase decision by selecting what most customers choose [54]. In marketing, brand popularity has been utilized as an advertising cue in order to stimulate consumer behavior positively [61]. The influence of popularity cues on behavior can be explained by social norm theory which attempts to understand social influences on an individual’s behavioral change [62]. How most people behave in a situation motivates an individual’s behavioral change by inducing a consumer to choose a particular brand that most consumers choose [54]. This supports the view that to determine what is right is to seek the approval of others [63] and justifies why consumers consciously look to other consumers when making a purchase decision [64]. In marketing research, it has been established that consumers being exposed to an advertisement using a popularity cue are more likely to have higher perceived quality, lower perceived risk [65], and higher intention to purchase the brand [61] compared to those being exposed to an advertisement without a popularity cue. Based on the theoretical and empirical foundations, this present study measures the extent to which brand popularity influences customers’ choice of restaurant. 

#### 2.1.5. Personal or Past Experience with a Restaurant

Past experience has been regarded as a key factor in customers’ post-consumption evaluations [66,67]. It is an important variable in understanding how consumer behavior is formed. The choice of brand does not affect repeat customers in the same way as first-time customers as there is an influence of previous experience in customers’ subsequent response to the purchase [68]. Common sense suggests that there is a high tendency of repeat patronage for repeat customers because they have visited the restaurant before and know what to expect on the next visit [69]. Furthermore, these two segments vary in their motives to consume products or services [70]. First-time customers may visit the restaurant for a new experience; repeat customers, on the other hand, revisit the restaurant to enjoy meals at a familiar place. Given this basis, we posit that personal (past) experience with the restaurant can be one of the most powerful situational factors that affect customers’ choice of restaurant.

#### 2.1.6. Variety of Menu Items

Restaurant consumers’ variety seeking behavior refers to the tendency to seek variety in their dining experiences [71]. The need for variety is based on individual’s prior purchase experiences which affect his/her choice in next purchase decision [72]. According to the theory of optimal stimulation level, consumers’ variety seeking behavior is triggered to reduce boredom from repeat purchases as well as to increase stimulation to the desired level [73]. Past studies suggested that the level of satiation or boredom varies depending on product/service attributes [74,75]. Consumers are likely to satiate on a product/service attribute if they relate the attribute to the primary feature being consumed [76]. For example, if cake is thought of as a food per se, then consumers tend to satiate on specific attributes (e.g., flavor, color, shape) and seek variety among the cakes. The attribute satiation model, proposed by McAlister [75], explains consumer choice behavior. It predicts choice behavior at a point in time; as product items decrease and are refilled, consumer’s product preference ranking, however, will likely change. To put it simply, boredom with certain product/service attributes (e.g., taste, color) may lead to variety seeking. Customers cognitively evaluate what they experience when eating-out at a restaurant [77]. Higher perceived variety leads to greater consumption [78]. In restaurant consumption, consumers’ need for variety can be satisfied in the offering of a variety of menu items. When choosing restaurants, consumers may choose one that offers a variety of menu options (although all the menu options are not eventually purchased). In these respects, we suggested that a variety of menu items is likely to be a crucial factor for those seeking variety in their dining experiences. 

#### 2.1.7. Menu Price

Price is a crucial marketing element in predicting consumer behavior in the restaurant industry [79]. It has been established as one of the highest-ranked factors for restaurant selections [80]. Consumers usually remember an objective or actual price to a certain extent that is meaningful to them, also known as perceived price [81]. Perceived price refers to what is given up, including monetary and non-monetary costs (e.g., money, time, and/or effort) to obtain a product or service [81]. The effect of price on consumer decision-making can be explained by the difference between reference price and actual price in product/service selection [82]. Reference price is compared against the actual price of a product/service in deciding whether or not to choose the product/service. An internal reference price (i.e., generated from past purchase experience) is a more important variable than an external reference price (i.e., generated from advertisement) in affecting consumers’ purchase behavior for regularly purchased product/service categories, such as meals in restaurants [83]. In restaurant settings, perceived price is commonly operationalized as meal price for which a customer transacts during his/her dining at a restaurant [84]. It has been established that consumers use price to evaluate the service quality as it partially acts as a clue for the quality [85]. Consequently, we measure the extent to which menu price influences customers’ choice of restaurant.

#### 2.1.8. Sales Promotion

Sales promotion creates a monetary incentive to purchase by reducing price for a certain quantity or increasing quantity for the same price [85]. It is a strategy that marketers offer to customers to satisfy their financial needs [86]. Marketers often employ sales promotion to encourage repeat purchase, induce product trials, or promote brand switching behavior [87]. Sales promotion provides customers with immediate financial incentives [88], but it may put a brand at risk by moving customers’ attention away from quality to a temporary financial incentive [89]. In fact, sales promotion appeals to price sensitive consumers who are willing to sacrifice quality for price or see all products in a certain product category as being equal [90]. Given that sales promotion is a common promotional strategy for attracting customers and generating revenue immediately in the foodservice industry [91], such as in restaurants, it is important to measure how it is likely to affect customers’ selection of restaurant.

#### 2.1.9. Location

Location has been well-identified as a strategic success factor for a restaurant business to stay competitive in the industry [92,93]. A strategic restaurant location can attract more customers to the restaurant, provide convenience to customers, and has a positive effect on customer loyalty [94]. Restaurants use location strategy to cater to target market/s and enhance the restaurant visibility [95]. For consumers, restaurant selection is dependent not only on location but also restaurant characteristics such as type of food served, facilities, size, etc. [69]. Nevertheless, given that location determines customer access to particular products or services, it remains fundamental to the decision-making of customers and is paramount to the success of a restaurant operation [96,97]. Consequently, this study determines the degree to which location shapes the restaurant customer decision-making process. 

### 2.2. Eating-out Occasions (Quick Meal/Convenience, Social Occasion, Business Necessity, and Celebration) and Customer Behaviors

Customers seek dining consumption experiences for different reasons [25]. As dining consumption occasions drive customer behavior, it is reasonable to assume that customers’ choice of restaurant is influenced by dining-out occasions. Past research has indicated that dining occasions influence customer choice in the restaurant selection process. One example of this can be found in a study by Kivela [98] which examined dining occasions (i.e., celebration, business, social, and convenience/quick meal) in understanding customers’ restaurant choice. The findings revealed that location was most related to convenience/quick meal occasion; food quality was perceived to be important for celebration and business occasions; and cleanliness seemed to be one of the important factors in customer choice of restaurant. In a similar vein, Ponnam and Balaji [25] investigated visitation motives (in place of dining occasions) and restaurant attributes in casual dining restaurants. Customers were found to have different motives (i.e., dine out, celebration, hang out, take-away, and date) for patronizing a casual dining restaurant. More specifically, dine out and take-away motives were found to be highly related to gourmet taste, celebration motive was strongly associated with hospitality service, hang-out motive was related to staff responsiveness, and date motive was highly correlated with ambiance and staff responsiveness. Overall, restaurant customers have specific reasons for patronizing specific types of restaurants.

### 2.3. Restaurant Types (Full-Service, Quick-Casual, and Quick-Service) and Customer Behaviors

Every restaurant provides three basic attributes (i.e., food, service, and physical environment) to customers. Each type of restaurant has its distinct attributes to differentiate the restaurant’s characteristics from the other restaurant types and to appeal to its target market [3,77]. Customers expect a certain level of quality according to the attributes provided by restaurants [99]. In the present study, restaurant services are categorized into three types: full-service, quick-casual, and quick-service [100]. A quick-service restaurant accentuates convenience and efficiency, such as low food price, quick service, convenient location, long hours of operation, and drive-through service [101]. Food is prepared in a standardized process that can be distributed immediately for ordering and consumption [100]. Customers visiting fast food restaurants are predominantly concerned about convenience when eating-out [3]. Quick-casual dining restaurant, a limited-service dining style, serves moderately-priced food in a casual dining atmosphere [100]. It is less expensive than a full-service restaurant but serves more high-quality food than a quick-service restaurant. Food is made-to-order and innovative food may be served to cater for sophisticated tastes. Quick-casual restaurants attract customers by serving good quality food at a reasonable price in a relaxed atmosphere [102]. A full-service restaurant provides meal courses and professional services by well-trained staff in an upscale or midscale dining atmosphere [98]. Full-service restaurants appeal to customers who consider emotional value to be an important factor when dining-out [3]. 

## 3. Methodology

### 3.1. Measures

A self-administered questionnaire was designed to measure the key factors importance, dining occasions, restaurant segments, and demographics. The first section of the questionnaire measured respondent’s eating-out information: type of restaurant and eating-out occasion. The second section comprised of key factors in restaurant selection: word-of-mouth recommendations from people I know, online reviews from customers (e.g., through Facebook, Twitter, blogs, TripAdvisor, etc.), brand reputation, brand popularity, personal (or past) experience with the restaurant, variety of menu items, price, sales promotion, and location. The respondent was asked to rank the factors from 1 (the most important) to 9 (the least important) when he/she chooses a restaurant. The factors were identified from an extensive review of past studies pertaining to restaurant management [4,5,8,25,69,103,104]. Then, we refined the factors through formal discussions with three academic professionals in restaurant management. Based on the discussions, “word-of-mouth recommendations”, “online reviews”, and “sales promotion” were further detailed. “Word-of-mouth recommendations” was specified as “word-of-mouth recommendations from people I know”; “online reviews” was rephrased as “online reviews from customers (e.g., through Facebook, Twitter, blogs, TripAdvisor, etc.)”; and “sales promotion” was specified with examples—“sales promotion (e.g., discounts, happy hours)”. The third section contained questions about basic demographics, such as gender, age, occupation, personal monthly net income, and level of education attainment.

### 3.2. Sample and Data Collection

We employed a descriptive survey research design to achieve the research purpose. A pencil-and-paper survey was conducted in 2017. Individuals were approached at six shopping centers in Klang Valley, Malaysia. Klang Valley is home to a number of popular and major shopping centers located in the urban cities, which include Kuala Lumpur and Petaling Jaya [105]. Every one of the shopping centers has a collection of stores, including local and international restaurant brands. Our trained research enumerators selected individuals through a convenience sampling method. Potential participants were politely approached in public seating areas at the shopping centers. To ensure that the individuals were qualified to participate in this survey, three screening questions were asked:

1. Do you regularly eat-out at restaurants on weekends?
2. What is your age?
3. Are you currently employed/working?

The individuals who regularly eat-out on weekends, aged 25 years and older, and were currently working were invited to participate in this anonymous survey. This group of individuals was selected because we believed that this group of respondents was capable of earning a disposable income and making decisions in restaurant selection. It has been reported that employed and educated consumers seem to seek variety in product/service decision-making [106]. Furthermore, eating-out has become prevalent among urban consumers in Malaysia [107]. We did not consider weekdays as eating-out on weekdays might not be a volitional behavior given that people are usually occupied with their daily work routine, and thus restricting their decision in choosing a restaurant. Every respondent was presented with a short statement recalling the experience of eating-out. The statement was described as follows: “*Think of your most recent visit to a restaurant in the past three months. It is a different kind of restaurant (that may have a distinctive feature such as menu, restaurant ambiance, or service style) from the ones that you commonly patronize. You made the decision to go to the restaurant*”. Respondents then indicated the type of restaurant and the dining occasion for the restaurant visit. They were also asked to provide rankings of the key factors in the restaurant selection decision. Lastly, respondents were asked to fill out the demographics section in the questionnaire. 

The survey questionnaires were distributed to a total of 617 restaurant customers. After eliminating unusable responses among the completed responses, 539 responses were coded for data analysis. More than half of the respondents were females (54.6%). The majority of the respondents were in the age range of 25 to 44 years old (80.9%), had a personal monthly net income of MYR 2000 to MYR 5999 (68.4%), and obtained a tertiary education (50.7%). This reflects Malaysia’s population which was relatively young and educated [107]. With regards to occupation, about 27.3% held executive/managerial/administrative position and about 22.4% were self-employed.

## 4. Results

### 4.1. General Order of Importance for Restaurant Choice

**Figure 1:**
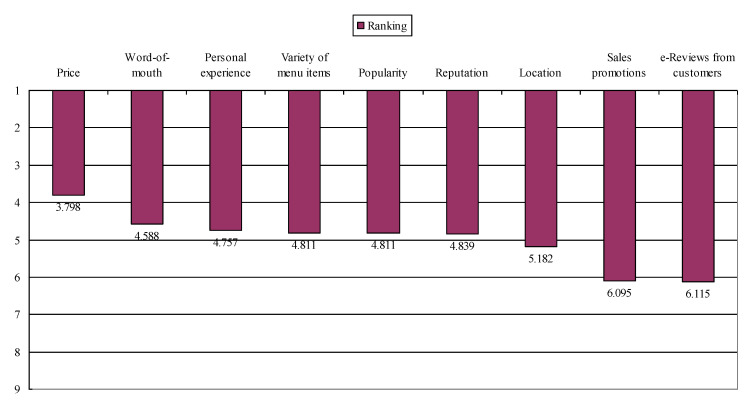
Overall ranking. The most/least important factor when choosing a restaurant. The value “1” indicates the most important criteria to consider when choosing a restaurant, and the value “9” indicates the least important criteria. Thus, the figure shows that “price” which is closer to “1” as compared to other variables is ranked the most important factor that patrons consider when selecting a restaurant.

The order of criticality among the factors that are vital for patrons’ restaurant selection (i.e., word-of-mouth recommendations from people I know, online reviews from customers, reputation, popularity, personal (or past) experience with the restaurant, variety of menu items, price, sales promotion, and location) was examined. Using IBM SPSS Statistics 20 (IBM, New York, NY, USA), a descriptive analysis was conducted based on the rank that the survey participants indicated. Table 1 and Figure 1 present the results of the analysis. As noted, the value “1” indicates the most important criteria to consider when choosing a restaurant, and the value “9” indicates the least important criteria. Thus, the results show that “price” which is closer to “1” as compared to other variables is ranked the most critical thing that patrons consider when choosing a restaurant. 

This finding implies that when making a decision to select a restaurant, patrons consider price as the most important factor, word-of-mouth from people they know as the second most important factor, personal/past experience as the third most important factor, variety of menu items as the fourth important factor, popularity as the fifth important factor, reputation as the sixth important factor, location as the seventh important factor, sales promotion as the eighth important factor, and online reviews from customers as the least important factor in sequence. In addition, about 25% of the participants ranked price as “1”. About 17.3%, 14.5%, 8.3%, 9.3%, 9.1%, 8.9%, 3.3%, and 4.3% ranked word-of-mouth, personal experience, variety of menu items, popularity, reputation, location, sales promotion, and online reviews from customers as “1”, respectively. Meanwhile, about 6.3% of the participants ranked price as “9”. In addition, about 8.3%, 8.5%, 9.5%, 6.7%, 6.5%, 13.0%, 17.6%, and 23.7% ranked word-of-mouth, personal experience, variety of menu items, popularity, reputation, location, sales promotion, and online reviews from customers as “9”, respectively. Table 2 further displays the significance of the restaurant choice factors ranking. The *t*-test results demonstrated that in general, price was significantly more important than word-of-mouth, and that location was significantly more important than sales promotion. This result contributed to achieving the first research objective of the present study. 

### 4.2. Ranking by Eating-out Occasions

**Figure 2:**
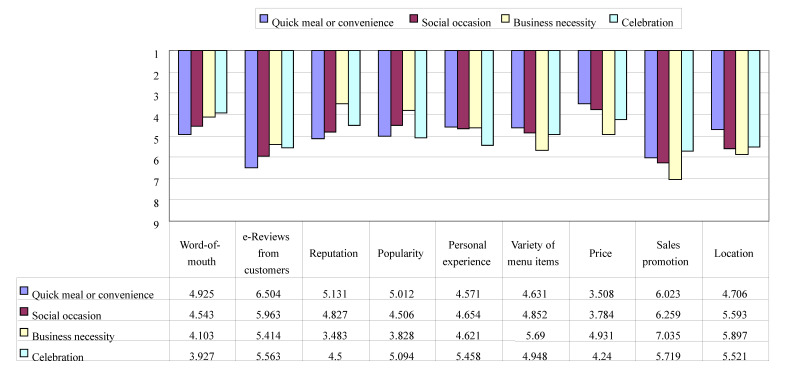
Ranking by eating-out occasions. 1: “The most important criteria to consider when choosing a restaurant”; 9: “The least important criteria to consider when choosing a restaurant”. Quick meal/convenience (*n* = 252), Social occasion (*n* = 162), Business necessity (*n* = 29), Celebration (*n* = 96).

The order of importance among restaurant choice factors by customers’ eating-out occasions (i.e., quick meal/convenience, social occasion, business necessity, and celebration) was examined by using a descriptive analysis. The details are shown in Figure 2. The top three restaurant choice factors in the occasion of quick meal/convenience were price (mean = 3.508, SD = 2.476), personal/past experience (mean = 4.571, SD = 2.610), and variety of menu items (mean = 4.631, SD = 2.427). In the case of social occasion, price (mean = 3.784, SD = 2.531), popularity (mean = 4.506, SD = 2.420), and word-of-mouth (mean = 4.543, SD = 2.659) were ranked as the three major choice factors. In the occasion of business necessity, unlike the previous two occasions, reputation (mean = 3.483, SD = 2.064) was ranked in the first place, followed by popularity (mean = 3.828, SD = 2.019), and word-of-mouth (mean = 4.103, SD = 2.440). Lastly, in the occasion of celebration, the top three restaurant selection factors were word-of-mouth (mean = 3.927, SD = 2.580), price (mean = 4.240, SD = 2.615), and reputation (mean = 4.500, SD = 2.362).

Table 3 discloses the differences in importance of restaurant choice factors across eating-out occasions. The one-way ANOVA findings indicated that while variety of menu items was not statistically significant, the importance of word-of-mouth, online review from customers, reputation, popularity, personal experience, price, sales promotion, and location were statistically significant across eating-out occasions. The non-significant difference in variety of menu items across eating-out occasions suggests that the attribute is equally important for all the occasions. This is consistent with Kivela et al. [69] where variety of menu was a crucial attribute determining customer evaluation of restaurant experience. A closer examination of the ranking by eating-out occasions further indicated that word-of-mouth was particularly crucial in celebration, followed by business necessity, social occasion, and quick meal/convenience. In addition, online reviews from customers were critical in the order of business necessity, celebration, social occasion, and quick meal/convenience. Reputation was especially important in business necessity, followed by celebration, social occasion, and quick meal/convenience. Popularity was particularly critical in business necessity, followed by social occasion, quick meal/convenience, and celebration. Moreover, personal experience was important in the order of quick meal/convenience, business necessity, social occasion, and celebration. Price was crucial in quick meal/convenience, social occasion, celebration, and business necessity in sequence. Sales promotion was important in the order of celebration, quick meal/convenience, social occasion, and business necessity. Further, location was particularly critical in the occasion of quick meal/convenience, followed by celebration, social, and business necessity. This result contributed to achieving the second research objective of this study. 

### 4.3. Ranking by Restaurant Types

**Figure 3:**
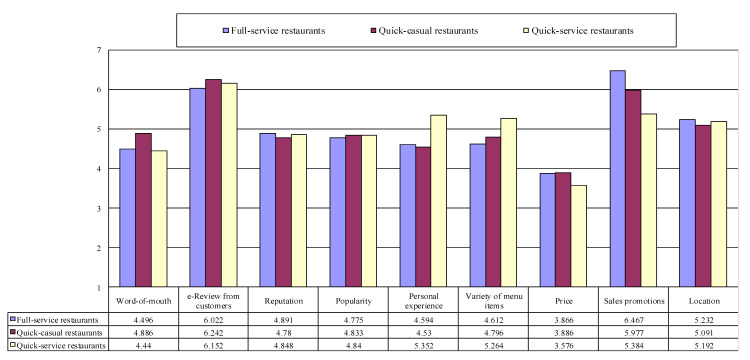
Ranking by restaurant types. 1: “The most important criteria to consider when choosing a restaurant”; 7: “The least important criteria to consider when choosing a restaurant”. Full-service restaurants (*n* = 276), Quick-casual restaurants (*n* = 132), Quick-service restaurants (*n* = 125).

Ranking by restaurant types (i.e., full-service restaurants, quick-casual/convenience restaurants, and quick-service restaurants) was investigated by using a descriptive analysis. First, the order of criticality among the nine choice factors for full-service restaurants was examined. The results are exhibited in Figure 3. Our finding indicated that price (mean = 3.866, SD = 2.436) was ranked in first place, followed by word-of-mouth (mean = 4.496, SD = 2.632), personal experience (mean = 4.594, SD = 2.654), variety of menu items (mean = 4.612, SD = 2.415), popularity (mean = 4.775, SD = 2.415), reputation (mean = 4.891, SD = 2.384), location (mean = 5.232, SD = 2.576), online reviews from customers (mean = 6.022, SD = 2.401), and sales promotion (mean = 6.467, SD = 2.325). This finding implies that when choosing a full-service restaurant for eating out, customers consider the above order in sequence. 

Second, the order of importance among the choice factors for quick-casual restaurants was examined. While price (mean = 3.886, SD = 2.811) was found as the most critical factor, the order of the rest of the factors in quick-casual restaurants was little different from that of the full-service restaurants. Our results revealed that personal experience (mean = 4.530, SD = 2.260), reputation (mean = 4.780, SD = 2.590), variety of menu items (mean = 4.796, SD = 2.291), popularity (mean = 4.833, SD = 2.282), word-of-mouth (mean = 4.886, SD = 2.754), location (mean = 5.091, SD = 2.593), sales promotion (mean = 5.977, SD = 2.352), and online reviews from customers (mean = 6.242, SD = 2.542) were the second, third, fourth, fifth, sixth, seventh, eighth, and ninth important factors in sequence when customers select a quick-casual restaurant.

Lastly, we examined the rank indicated by customers when making a decision for selecting a quick-service restaurant. In the case of quick-service restaurant choice, participants ranked price (mean = 3.576, SD = 2.547) as the most crucial thing that they consider among the nine factors driving restaurant selection, followed by word-of-mouth (mean = 4.440, SD = 2.775), popularity (mean = 4.840, SD = 2.329), reputation (mean = 4.848, SD = 2.279), location (mean = 5.192, SD = 2.678), variety of menu items (mean = 5.264, SD = 2.609), personal experience (mean = 5.352, SD = 2.515), sales promotion (mean = 5.384, SD = 2.327), and online reviews from customers (mean = 6.152, SD = 2.393). The results pertinent to the ranking among important restaurant choice factors by restaurant types contributed to achieving the third research objective of the present study. 

Table 4 further illustrates the differences in importance of restaurant choice factors across restaurant types. The one-way ANOVA findings revealed that the importance of personal experience, variety of menu items, and sales promotion were statistically different across restaurant types. Personal experience was important in the order of quick-casual, full-service, and quick-service. Variety of menu items was crucial in full-service, quick-casual, and quick-service in sequence. Sales promotion was important in the order of quick-service, quick-casual, and full-service. The insignificant difference in price implies that price is the most critical factor for all the three types of restaurant, which supports the aforementioned discussion.

## 5. Discussion

Faced with the complex phenomenon of eating-out, our study extends the body of knowledge on the relative importance of restaurant selection criteria. Our investigation into customers’ perceived importance of restaurant selection factors and how they vary across situational factors, namely dining occasions and restaurant segments, presents empirical evidence regarding customers’ choice of restaurant. Our study provides three insights. First, menu price was perceived as the most important criterion in all nine criteria when consumers choose a restaurant to eat-out. This is not surprising as since the Malaysian government imposes the implementation of a 6% Goods and Service Tax (GST) in 2015, consumers are becoming more price-sensitive and cautious about spending on eating-out [108]. Another plausible reason is that, consistent with past research advocating the salient role of price as a clue of consumers’ expectation and evaluation of product or service performance [109,110], our findings suggest that menu price has the overall greatest importance for restaurant customers. The role of price in influencing restaurant customers’ decision-making could be attributed by the common belief that price has been used as a reference in making quality inference [84].

Second, our study ranked the level of importance among the factors for customers to consider when choosing a restaurant by eating-out occasions. The importance level of menu price was greatest for both quick meal/convenience and social occasion; brand reputation was the most important for business necessity; and word-of-mouth recommendation (from the people I know) was greatest for celebration. On the other hand, online reviews carried the least importance for quick meal/convenience, and sales promotion was ranked being the least important for social occasion, business necessity, and celebration. Our findings provide empirical evidence that eating-out occasion is the key determinant of restaurant selection criteria. This supports the assertion that restaurant customers have distinctive reasons when patronizing restaurants [25,27,98]. The findings of this study allow restaurant selection criteria to be segmented in relation to their primary use occasion.

Third, our study investigated the relative importance among the restaurant selection factors by restaurant types. Menu price was perceived as being the most important criterion and sales promotion was the least important criterion for full-service restaurants. Menu price was also ranked highest on quick-casual restaurant selection criteria and online review was perceived to be the least important. The nature of our sample might shed light on the prevalence of quick-casual units in Malaysia. The majority of the respondents in this study were young working adults and middle-income consumers. This group of consumers prefer an informal and comfortable environment as well as reasonably-priced menu items [108]. Similarly, menu price was ranked highest and online review was ranked lowest on quick-service restaurant selection criteria. The substantial growth of the restaurant market in Malaysia and the homogeneity of offerings across restaurants within one segment might shed light on the importance of menu price in customers’ choice of restaurant. Customers have too many choices of restaurant when it comes to eating-out. Our study suggests that when there is a huge number of restaurant options with similar product or service offerings, there is a greater tendency for customers to rely on the prices when making decision. Thus, it is not surprising that customers are relatively mindful of prices when making eating-out decisions. This is consistent with Lewis’s [111] argument that price is a key factor in differentiating within a set of product class.

## 6. Implications

The restaurant industry is highly competitive. The understanding about restaurant customer behavior is vital for restaurants to achieve a sustainable restaurant business growth. Several managerial implications emerge from our study. First, restauranteurs should be alert to the comparative importance of factors in customer decision making. Such importance levels may trigger restauranteurs to consider marketing strategies for their restaurant that they may not have otherwise considered. For example, considering our finding that menu price is customers’ top priority in restaurant selections for full-service, quick-casual, and quick-service restaurants, when food is priced appropriately, it can positively influence customers’ decision. Customers encode menu price as a synopsis of dining experience. The price perception is influential in assisting customers make a choice, suggesting the need to adopt effective pricing strategies. Restauranteurs should utilize the principle of integrated marketing communication strategies and grasp every opportunity to manage customer perception of price. Rather than leaving customer perception of price to chance, restauranteurs can take a proactive role in setting up value-based pricing strategies. For example, quick-service restauranteurs should consider implementing the practice of several international fast-food chains who regularly remind customers of their meal savings. When creating a pricing strategy, quick-casual restauranteurs should keep in mind that their customers value good quality food at a reasonable price in a comfortable dining atmosphere. The pricing strategies of full-service restauranteurs should appeal to customers who appreciate emotions in dining experiences as they typically seek a dining experience beyond eating, therefore strengthening competitive price perception. Quick-service and full-service restauranteurs must get customers to recognize the eating-out benefits they receive for the price they pay. In other words, the advertising messages should highlight the benefits of eating in the restaurants relative to the prices.

Second, word-of-mouth recommendation (from the people I know), which was ranked second in the important factors list, can strengthen customers’ decision to choose a restaurant. In the restaurant industry, word-of-mouth recommendation is influential, and most importantly, it costs a restaurant nothing to promote its products/services to potential customers. Thus, we suggest that restaurateurs consistently provide high-quality products and services to trigger positive word-of-mouth. Achieving customer satisfaction stimulates positive communications in a customer’s direct contacts and immediate surroundings [112]. Third, personal experience, which was ranked third in the important factors list, can affect restaurant customer decision-making. Most Malaysian consumers are well-informed and sophisticated, and they appreciate quality in dining experience [108]. If a restaurant receives favorable evaluations of their dining experience in the restaurant from existing customers, the positive evaluations can have a considerable impact on customer satisfaction and, consequently, on their behavioral intentions, such as revisit intentions [113].

Forth, a closer look into the relative important of restaurant selection criteria across eating-out occasions shows that restaurant customers rated the importance level of restaurant selection criteria differently according to eating-out occasions. As the restaurant selection criteria are influenced by the eating-out occasions, we suggest that decisions relating to personalizing the promotional strategies should be undertaken. Because customers attach different levels of importance to restaurant selection criteria, it is essential to tailor distinctive efforts for optimal effects on restaurant customer behavior. Promotional tactics should reflect the consistency between purpose of eating-out and restaurant selection factors. Menu prices are critically important when customers patronize a restaurant for quick meal/convenience and social occasion. In Malaysia, with growing urbanization and changing lifestyles, an increasingly great number of consumers seek convenience through eating-out. Financial incentives (such as value meals and set meals) and psychological pricing (such as 9-ending prices) are thus recommended for customers visiting a restaurant for quick meal/convenience or social occasions. Restaurant reputation is vital when customers choose a restaurant for business necessity. Customers may expect to have good food and drink in a comfortable physical environment to entertain their business clients. Restauranteurs should maintain the standards of these attributes to meet the needs and wants of their customers. Word-of-mouth is essential when customers select a restaurant for celebration occasion. Considering that customers visiting a restaurant to celebrate a special occasion (e.g., birthday, wedding anniversary), it is important for customers to choose the right restaurant where they can happily cherish the special moment. Accordingly, restauranteurs should increase their competitive advantage by creating customer engagement opportunities, such as sharing dining experiences on social media networks and facilitating customer-to-customer interactions.

## 7. Limitations and Recommendations for Future Research

There are several limitations to this study that should be addressed for future research. First, we conducted data collection in only one area in Malaysia (i.e., Klang Valley), thus limiting the generalizability of the conclusions. Other metropolitan cities across countries may be studied to obtain comparative results. Second, how respondents evaluate the difference in important ranking was not examined. In other words, the variables, such as values associated with eating-out, that may have a significant influence on important factor ratings should be further examined. More theoretical and practical implications regarding customers’ perceived importance of restaurant selection factors could be drawn when the underlying variables explaining the outcomes are investigated. Third, this study identified the nine factors based on the existing empirical studies on consumer behavior in the restaurant industry. The importance of certain restaurant choice factors, which included word-of-mouth, online reviews, reputation, popularity, price, and location were not statistically different between full-service, quick-casual, and quick-service restaurants (Table 4). This suggests that these factors are equally important for all the three types of restaurant. Given the fact that consumer decision-making in restaurant selection is dynamic and may be driven by emerging factors or reasons, future research is suggested to delve into this topic by utilizing qualitative methods. Fourth, this study was descriptive in nature, thus failing to include delicate statistical techniques and to suggest a causal model of the antecedents and consequence of customers’ decision. The contribution of this study could be strengthened through more robust quantitative research approach efforts. Fifth, the subgroups (i.e., quick meal, social occasion, business, celebration) have different number of sample size. Future research should balance the sample size for these subgroups. In addition, future research should increase the sample size to effectively compare the constituents of eating-out occasions.

## 8. Conclusions

Customer expectations of restaurant offerings are ever-increasing, and they are now more demanding in choosing better restaurant choices based on what they can get from their decision. An investigation of key factors driving customers’ restaurant choice in eating-out decision making not only can help restaurateurs understand restaurant customer perception of key factors when selecting a restaurant, but also form appropriate marketing strategies to attract existing and potential customers and outperform competitors. Faced with the complex phenomenon of eating-out, our study extends the body of knowledge on the relative importance of restaurant selection criteria. Our investigation into customers’ perceived importance of restaurant selection factors and how they vary across situational factors, namely dining occasions and restaurant segments, presents empirical evidence regarding customers’ choice of restaurant. Our study has three important findings. First, menu price is perceived as the most important criterion in all nine criteria (i.e., word-of-mouth, online customer review, brand reputation, brand popularity, personal (past) experience, menu variety, menu price, sales promotion, and location) when consumers choose a restaurant to eat-out. Second, eating-out occasion is the key determinant of restaurant selection criteria. More specifically, the importance level of menu price is greatest for both quick meal/convenience and social occasion; brand reputation is the most important for business necessity; and word-of-mouth recommendation (from the people I know) is greatest for celebration. Third, menu price was perceived as being the most important criterion for full-service restaurants, quick-casual restaurants, and quick-service restaurants, respectively. This suggests that when there are a huge number of restaurant options with similar product or service offerings within a restaurant segment, there is a greater tendency for customers to rely on the prices when making decision. Overall, the findings of this study add to the restaurant management literature that customers’ restaurant choice is markedly affected by situational factors [69,98]. It is concluded that customers’ perceived importance of restaurant selection factors are important considerations in the occasion for which a restaurant is patronized and in the choice of restaurant type. The findings are valuable to restauranteurs in developing occasion-based and restaurant type-based segmentations based on restaurant selection factor priorities.

## Figures and Tables

**Table 1:** Overall ranking of restaurant choice factors.

Rank	Restaurant Choice Factors	Mean ± Std. Deviation	Skewness	Kurtosis
1	Price	3.798 ± 2.558	0.588	−0.887
2	Word-of-mouth	4.588 ± 2.692	0.168	−1.327
3	Personal/past experience	4.757 ± 2.551	0.040	−1.173
4	Variety of menu items	4.811 ± 2.442	0.172	−1.082
5	Popularity	4.811 ± 2.363	0.076	−1.041
6	Reputation	4.839 ± 2.402	0.018	−1.137
7	Location	5.182 ± 2.604	−0.053	−1.283
8	Sales promotion	6.095 ± 2.367	−0.497	−0.910
9	Online review from customers	6.115 ± 2.426	−0.419	−0.942

1: “The most important criteria to consider when choosing a restaurant”; 9: “The least important criteria to consider when choosing a restaurant”. Std. Deviation refers to Standard Deviation.

**Table 2:** Significance of restaurant choice factors ranking.

Restaurant Choice Factors	Mean ± Std. Deviation	*t*-Value	*p*-Value
Price	3.798 ± 2.558	−4.207 ***	0.000
Word-of-mouth	4.588 ± 2.692		
Word-of-mouth	4.588 ± 2.692	−0.962	0.336
Personal/past experience	4.757 ± 2.551		
Personal/past experience	4.757 ± 2.551	−0.349	0.727
Variety of menu items	4.811 ± 2.442		
Variety of menu items	4.811 ± 2.442	0.000	1.000
Popularity	4.811 ± 2.363		
Popularity	4.811 ± 2.363	−0.224	0.823
Reputation	4.839 ± 2.402		
Reputation	4.839 ± 2.402	−1.934	0.054
Location	5.182 ± 2.604		
Location	5.182 ± 2.604	−6.706 ***	0.000
Sales promotion	6.095 ± 2.367		
Sales promotion	6.095 ± 2.367	−0.125	0.901
Online review from customers	6.115 ± 2.426		

1: “The most important criteria to consider when choosing a restaurant”; 9: “The least important criteria to consider when choosing a restaurant”. *** *p* < 0.001.

**Table 3:** Differences in restaurant choice factors across eating-out occasions.

Restaurant Choice Factors	Occasions	Mean ± Std. Deviation	*F*-Value	*p*-Value
Word-of-mouth	Quick meal	4.925 ± 2.739	3.623 *	0.013
	Social occasion	4.543 ± 2.659		
	Business necessity	4.103 ± 2.440		
	Celebration	3.927 ± 2.580		
Online review from customers	Quick meal	6.504 ± 2.311	4.944 **	0.002
	Social occasion	5.963 ± 2.566		
	Business necessity	5.414 ± 2.147		
	Celebration	5.563 ± 2.410		
Reputation	Quick meal	5.131 ± 2.350	5.073 **	0.002
	Social occasion	4.827 ± 2.471		
	Business necessity	3.483 ± 2.064		
	Celebration	4.500 ± 2.362		
Popularity	Quick meal	5.012 ± 2.275	3.692 *	0.012
	Social occasion	4.506 ± 2.420		
	Business necessity	3.828 ± 2.019		
	Celebration	5.094 ± 2.488		
Personal/past experience	Quick meal	4.571 ± 2.610	3.012 *	0.030
	Social occasion	4.654 ± 2.370		
	Business necessity	4.621 ± 2.871		
	Celebration	5.458 ± 2.509		
Variety of menu items	Quick meal	4.631 ± 2.427	1.832	0.140
	Social occasion	4.852 ± 2.268		
	Business necessity	5.690 ± 2.647		
	Celebration	4.948 ± 2.661		
Price	Quick meal	3.508 ± 2.476	3.999 **	0.008
	Social occasion	3.784 ± 2.531		
	Business necessity	4.931 ± 2.815		
	Celebration	4.240 ± 2.615		
Sales promotion	Quick meal	6.024 ± 2.336	2.693 *	0.045
	Social occasion	6.259 ± 2.397		
	Business necessity	7.035 ± 1.426		
	Celebration	5.719 ± 2.549		
Location	Quick meal	4.706 ± 2.547	5.552 **	0.001
	Social occasion	5.593 ± 2.594		
	Business necessity	5.897 ± 2.730		
	Celebration	5.521 ± 2.550		

* *p* < 0.05, ** *p* < 0.01.

**Table 4:** Differences in restaurant choice factors across restaurant types.

Restaurant Choice Factors	Restaurant Types	Mean ± Std. Deviation	*F*-Value	*p*-Value
Word-of-mouth	Full-service	4.496 ± 2.632	1.153	0.316
	Quick-casual	4.886 ± 2.754		
	Quick-service	4.440 ± 2.775		
Online review from customers	Full-service	6.022 ± 2.401	0.395	0.674
	Quick-casual	6.242 ± 2.542		
	Quick-service	6.152 ± 2.393		
Reputation	Full-service	4.891 ± 2.384	0.095	0.909
	Quick-casual	4.780 ± 2.590		
	Quick-service	4.848 ± 2.279		
Popularity	Full-service	4.775 ± 2.415	0.045	0.956
	Quick-casual	4.833 ± 2.282		
	Quick-service	4.840 ± 2.329		
Personal/past experience	Full-service	4.594 ± 2.654	4.561 *	0.011
	Quick-casual	4.530 ± 2.260		
	Quick-service	5.352 ± 2.515		
Variety of menu items	Full-service	4.612 ± 2.415	3.092 *	0.046
	Quick-casual	4.796 ± 2.291		
	Quick-service	5.264 ± 2.609		
Price	Full-service	3.866 ± 2.436	0.645	0.525
	Quick-casual	3.886 ± 2.811		
	Quick-service	3.576 ± 2.547		
Sales promotion	Full-service	6.467 ± 2.325	9.493 ***	0.000
	Quick-casual	5.977 ± 2.352		
	Quick-service	5.384 ± 2.327		
Location	Full-service	5.232 ± 2.576	0.131	0.877
	Quick-casual	5.091 ± 2.593		
	Quick-service	± 2.678		

* *p* < 0.05, *** *p* < 0.001.
